# Regulated maturation of malaria merozoite surface protein-1 is essential for parasite growth

**DOI:** 10.1111/j.1365-2958.2010.07324.x

**Published:** 2010-08-24

**Authors:** Matthew A Child, Christian Epp, Hermann Bujard, Michael J Blackman

**Affiliations:** 1 Division of Parasitology, MRC National Institute for Medical ResearchMill Hill, London NW7 1AA, UK; 2 Department für Infektiologie-Parasitologie, Universitaetsklinikum HeidelbergIm Neuenheimer Feld 324, D-69120 Heidelberg, Germany; 3 Zentrum für Molekulare Biologie Heidelberg (ZMBH), Universitaet HeidelbergIm Neuenheimer Feld 282, D-69120 Heidelberg, Germany

## Abstract

The malaria parasite *Plasmodium falciparum* invades erythrocytes where it replicates to produce invasive merozoites, which eventually egress to repeat the cycle. Merozoite surface protein-1 (MSP1), a prime malaria vaccine candidate and one of the most abundant components of the merozoite surface, is implicated in the ligand–receptor interactions leading to invasion. MSP1 is extensively proteolytically modified, first just before egress and then during invasion. These primary and secondary processing events are mediated respectively, by two parasite subtilisin-like proteases, PfSUB1 and PfSUB2, but the function and biological importance of the processing is unknown. Here, we examine the regulation and significance of MSP1 processing. We show that primary processing is ordered, with the primary processing site closest to the C-terminal end of MSP1 being cleaved last, irrespective of polymorphisms throughout the rest of the molecule. Replacement of the secondary processing site, normally refractory to PfSUB1, with a PfSUB1-sensitive site, is deleterious to parasite growth. Our findings show that correct spatiotemporal regulation of MSP1 maturation is crucial for the function of the protein and for maintenance of the parasite asexual blood-stage life cycle.

## Introduction

Clinical malaria results from replication of asexual blood-stage forms of the malaria parasite in erythrocytes. The parasite divides within a parasitophorous vacuole (PV), forming a multinucleated schizont that eventually undergoes cytokinesis to produce daughter merozoites. These are released from the infected host cell in a process called egress, and rapidly bind to and invade a fresh host cell. Primary interactions between the merozoite and its target erythrocyte involve parasite surface proteins, the most abundant of which is a large (approximately 200 kDa) glycosyl phosphatidylinositol (GPI)-anchored protein called merozoite surface protein-1 (MSP1) (Holder *et al.*, 1992; Gilson *et al.*, 2006). In the case of the most dangerous malarial species, *Plasmodium falciparum*, MSP1 is expressed with two associated partner proteins called MSP6 and MSP7 (Holder *et al.*, 1985; Stafford *et al.*, 1994; 1996; Pachebat *et al.*, 2001; Trucco *et al.*, 2001; Kauth *et al.*, 2003; 2006;). All three proteins are synthesized during development of the intracellular merozoite as precursors, which assemble into a complex (referred to as MSP1/6/7) at the parasite plasma membrane. Just before egress, all three proteins in the complex are proteolytically modified by the action of a single subtilisin-like serine protease called PfSUB1, which is released into the PV lumen from specialized organelles called exonemes (Yeoh *et al.*, 2007; Koussis *et al.*, 2009). This so-called primary proteolytic processing of MSP1/6/7 involves cleavage of the MSP1 precursor (which exists in two major allelic forms in *P. falciparum*) into four or five fragments (Holder *et al.*, 1985; 1987; Lyon *et al.*, 1986; 1987; McBride and Heidrich, 1987), whereas both MSP6 and MSP7 are N-terminally truncated (Stafford *et al.*, 1996; Pachebat *et al.*, 2001; 2007; Trucco *et al.*, 2001). The resulting processed products remain non-covalently associated on the merozoite surface until egress and invasion of a fresh erythrocyte. During invasion, the bulk of the complex is completely shed from the free merozoite surface through the action of a second, membrane-bound subtilisin-like protease called PfSUB2, which is discharged onto the parasite surface from a set of apically disposed parasite organelles called micronemes (Blackman and Holder, 1992; Harris *et al.*, 2005). This secondary processing step takes the form of a single cleavage on the C-terminal side of a Leu residue within the juxtamembrane MSP1 sequence QG/DML↓NISQ (Blackman *et al.*, 1990). This sequence is completely refractory to cleavage by PfSUB1 (Koussis *et al.*, 2009), and we have previously hypothesized that this may be an important factor in the spatiotemporal regulation of the two processing steps; primary processing of the MSP1/6/7 complex is initiated by release of PfSUB1 from exonemes into the PV lumen just before egress, but secondary processing cannot be mediated by PfSUB1 so can only occur upon subsequent release of PfSUB2 from micronemes following egress. MSP1 has been extensively studied due to its potential as a vaccine candidate, as antibodies against it can prevent invasion *in vitro* and protect against blood-stage challenge *in vivo*[see Holder (2009) and Vekemans and Ballou (2008) for recent reviews]. Additionally, there is accumulating evidence that MSP1 has a crucial role in mediating adhesive interactions between the invading merozoite and host erythrocyte (Su *et al.*, 1993; Goel *et al.*, 2003; Li *et al.*, 2004; Boyle *et al.*, 2010). In support of this, gene knockout studies have shown that MSP1 (O'Donnell *et al.*, 2000; Drew *et al.*, 2004; Combe *et al.*, 2009), but not MSP7 (Tewari *et al.*, 2005; Kadekoppala *et al.*, 2008) or MSP6 (E. Knuepfer and A. Holder, pers. comm.), is indispensable in the asexual life cycle of the parasite. All *Plasmodium* species possess orthologues of MSP1, and the protein is subjected to a similar two-step proteolytic processing in all those species where the phenomenon has been examined (O'Dea *et al.*, 1995; Blackman *et al.*, 1996; Wiser *et al.*, 1997; Jennings *et al.*, 1998). Small molecules or antibodies that inhibit PfSUB1 activity or interfere with PfSUB2-mediated shedding of the MSP1/6/7 complex can prevent invasion (Blackman *et al.*, 1994; Fleck *et al.*, 2003; Koussis *et al.*, 2009), leading to suggestions that proteolytic maturation of MSP1 is essential for invasion. However, both PfSUB1 and PfSUB2 have substrates other than MSP1 (Howell *et al.*, 2003; Yeoh *et al.*, 2007; Koussis *et al.*, 2009), so the specific importance of MSP1 maturation and the biological significance of its spatiotemporal regulation is unknown.

Here, we have used a mutagenesis-based approach to address this question. We first show that maturation of *P. falciparum* MSP1 by PfSUB1 is an ordered process, in which the primary processing site closest to its C-terminal end (the 38/42 site) is cleaved last, irrespective of polymorphisms throughout the rest of the molecule. Second, we demonstrate that perturbation of the processing order by replacing the secondary processing site in MSP1 with a PfSUB1-sensitive sequence that is cleaved more efficiently than the 38/42 site, cannot be tolerated by the parasite. Our results provide the first genetic evidence that correct regulation of MSP1 processing is critical for the function of the protein, and for maintenance of the erythrocytic life cycle of the malaria parasite.

## Results

### Most primary processing sites in MSP1 are dimorphic

**Fig. 1:**
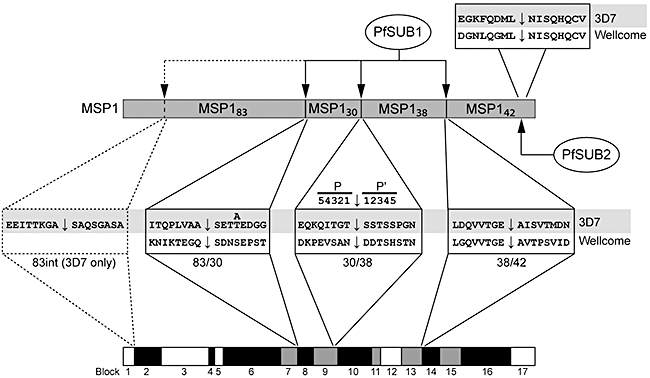
Most primary processing site sequences diverge between the two MSP1 allelic types.Primary proteolytic processing of MSP1 by PfSUB1 results in the production of the MSP1_83_, MSP1_30_, MSP1_38_ and MSP1_42_ fragments (top centre). The amino acid sequences within which cleavage occurs are shown for the two major dimorphic forms of MSP1, typified by those of the 3D7 and Wellcome isolates (centre). Also shown is an additional cleavage site (called 83int, indicated by dotted lines) that occurs only in the 3D7-type MSP1. The only detected microheterogeneity in the cleavage sites is a Thr-to-Ala substitution in the P4′ position of the 3D7-type 83/30 cleavage site. The positions of the primary cleavage sites relative to the distribution of conserved (open), variable (black) and semi-conserved (grey) blocks within MSP1, numbered 1–17 according to Tanabe *et al.* (1987) are shown (bottom). The sequence at which PfSUB2-mediated secondary processing occurs is also shown (top right). Note that PfSUB1 cannot cleave C-terminal to a Leu residue, and the secondary processing site is completely refractory to PfSUB1 (Withers-Martinez *et al.*, 2002; Yeoh *et al.*, 2007; Koussis *et al.*, 2009).

Like many blood-stage malarial surface proteins, *P. falciparum* MSP1 is highly polymorphic. Extensive early work showed that the protein can be divided into 17 regions or blocks of variable, less variable (semi-conserved) and conserved (non-polymorphic) sequence (Tanabe *et al.*, 1987). Polymorphism in the variable regions results from limited recombination between genes encoding two major dimorphic forms of MSP1, typified by those expressed by the 3D7 and Wellcome *P. falciparum* isolates (Tanabe *et al.*, 1987; 2007; Miller *et al.*, 1993). A third relatively uncommon MSP1 form, initially identified in the RO-33 parasite isolate, entirely lacks block 2 (which is composed of variable Ser–Xaa–Xaa tripeptide repeats) but is otherwise similar to the 3D7 type (Certa *et al.*, 1987; Peterson *et al.*, 1988; Tanabe *et al.*, 2007). Previous work by us (Blackman *et al.*, 1991; Cooper and Bujard, 1992; Stafford *et al.*, 1994; Koussis *et al.*, 2009) and others (Heidrich *et al.*, 1989) has precisely mapped all the primary processing sites in both the 3D7 and Wellcome types of MSP1. To examine the degree of amino acid conservation around these positions, sequences flanking the cleavage sites in all available full and partial *P. falciparum* MSP1 sequences deposited in PlasmoDB (http://plasmodb.org/plasmo/) and GenBank (http://www.ncbi.nlm.nih.gov/) were examined by multiple alignment. Sequences from a total of 35 complete (29 3D7-type and six Wellcome-type) and 130 partial MSP1 sequences were incorporated into the analysis. This allowed us to make two main observations regarding the sites. First, although all the cleavage sites (aside from the 3D7 type-specific 83int cleavage) are positionally conserved within the MSP1 sequences, all lie within semi-conserved or variable regions of the protein. However, within each form of MSP1, the alignments reveal no microheterogeneity in the residues closely flanking the cleavage sites, aside from the presence in just two deposited sequences of a Thr-to-Ala substitution at the P4′ position of the 83/30 cleavage site in the 3D7 form (Fig. 1). Our second observation was that of all the primary processing sites, only the 38/42 site (i.e. that closest to the C-terminus of MSP1) shows any significant identity between the dimorphic MSP1 forms. At this site, all four non-prime side amino acid residues (positions P4–P1 in Schechter and Berger nomenclature: Schechter and Berger, 1967) that are usually important for substrate recognition by subtilisin-like proteases (Siezen and Leunissen, 1997), plus the P5, P6 and P1′ residues, are identical between the MSP1 forms.

Earlier work on PfSUB1 substrate specificity defined a consensus PfSUB1 recognition motif of Ile/Leu/Val/Thr/Phe–Xaa–Gly/Ala–Paa(not Leu)↓Xaa (where Xaa is any amino acid residue and Paa tends to be a polar residue), plus a tendency for acidic residues or Ser or Thr at one or more of the proximal five positions on the prime side of the scissile bond (Koussis *et al.*, 2009). Beyond this, the requirements for optimal substrate cleavage by PfSUB1 are unknown. All the *P. falciparum* MSP1 primary processing sites fit this consensus, but as different amino acid target sequences are usually recognized with different affinities by proteases, our observations led us to formulate two hypotheses: first, that the different cleavage sites within MSP1 are likely to be cleaved by PfSUB1 at different relative rates; and second, that the 38/42 site may be of special significance in the processing pathway. Our next experiments focused on testing these predictions.

### Primary processing of MSP1 is ordered

To examine the relative rates of cleavage at the various MSP1 primary processing sites we exploited a modification of a previously used assay (Koussis *et al.*, 2009) in which protease inhibitor-treated, permeabilized schizonts containing predominantly unprocessed MSP1 precursor are exposed *in vitro* to exogenously added recombinant PfSUB1 (rPfSUB1). This assay is designed to mimic authentic primary processing, using the native, GPI-anchored, parasite-derived MSP1 as a substrate. To follow the course of processing over time, digests were sampled at intervals following addition of rPfSUB1 and examined by Western blot using monoclonal antibodies (mAb) or polyclonal antibodies specific for epitopes within both the MSP1_83_ and MSP1_42_ processing products.

**Fig. 2:**
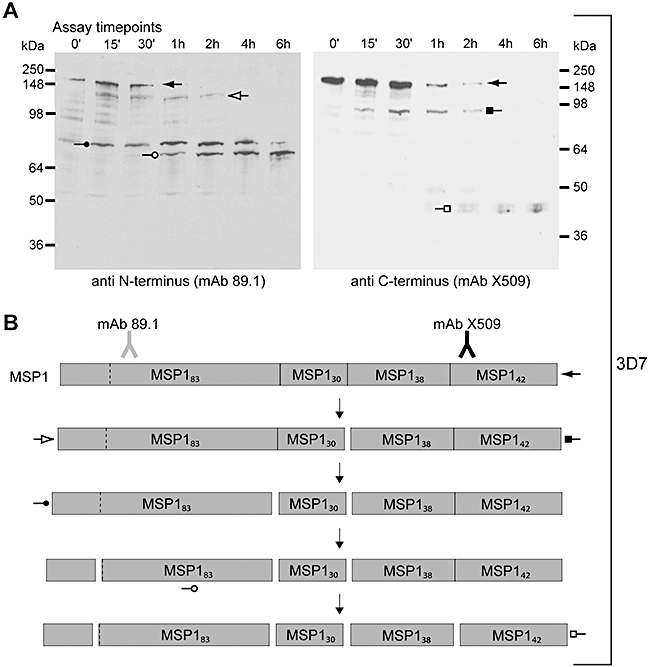
PfSUB1-mediated proteolytic processing of the 3D7 MSP1 is ordered.A. Typical time-course of digestion of native 3D7 *P. falciparum*-derived MSP1. Saponin-permeabilized 3D7 schizonts were incubated at 37°C in the presence of rPfSUB1. Samples taken at intervals were analysed by Western blot, probing with either the MSP1_83_-specific mAb 89.1 (left-hand side), or the MSP1_42_-specific monoclonal antibody X509 (right-hand side). For both blots the MSP1 precursor is indicated with a black arrow, processing intermediates are marked with an open arrow, or closed round or square-head lollipop, while terminal processing products are marked with an open round or square-head lollipop. No processing was observed in the absence of added rPfSUB1 (not shown).B. Schematic of the order of 3D7-type MSP1 primary processing, determined from the digestion assay shown in (A). Individual processed species are marked with arrow or lollipop symbols to aid comparison with the corresponding bands on the blots.

Figure 2 shows the results of a typical processing assay using schizonts expressing the 3D7-type MSP1. Processing was observed to proceed to the final products through two transient high molecular weight intermediates, one of which (approximately 120 kDa) was recognized by mAb 89.1 but not mAb X509 (Fig. 2A left-hand side), and the other of which (approximately 90 kDa) was recognized by mAb X509 but not mAb 89.1 (Fig. 2A right-hand side). These observations can only be accommodated by a model in which cleavage occurs first at the central 30/38 site (Fig. 2B). Subsequent to this, the MSP1_83_ product, followed by its N-terminally truncated form (both detected by mAb 89.1), appeared rapidly compared with the relatively slow rate of appearance of the final MSP1_42_ product detected by mAb X509. This led us to conclude that cleavage at the 38/42 site is slow relative to cleavage at all the other sites.

To independently test this interpretation, we next examined processing by rPfSUB1 of a full-length recombinant form of 3D7 MSP1 (*Supporting information* Fig. S1). Once again, probing the blots with mAb 89.1 or mAb X509 showed that processing progressed through two different-sized high molecular weight intermediates, each of which was recognized by only one of the two mAbs, indicating that the first cleavage occurs at the central 30/38 site. Cleavage then occurred at the 83/30 site, followed by cleavage at the 83int site. However, once again conversion of the mAb 89.1-reactive intermediate to smaller fragments was rapid compared with breakdown of the X509-reactive intermediate, again suggesting that the 38/42 site is processed last.

**Fig. 3:**
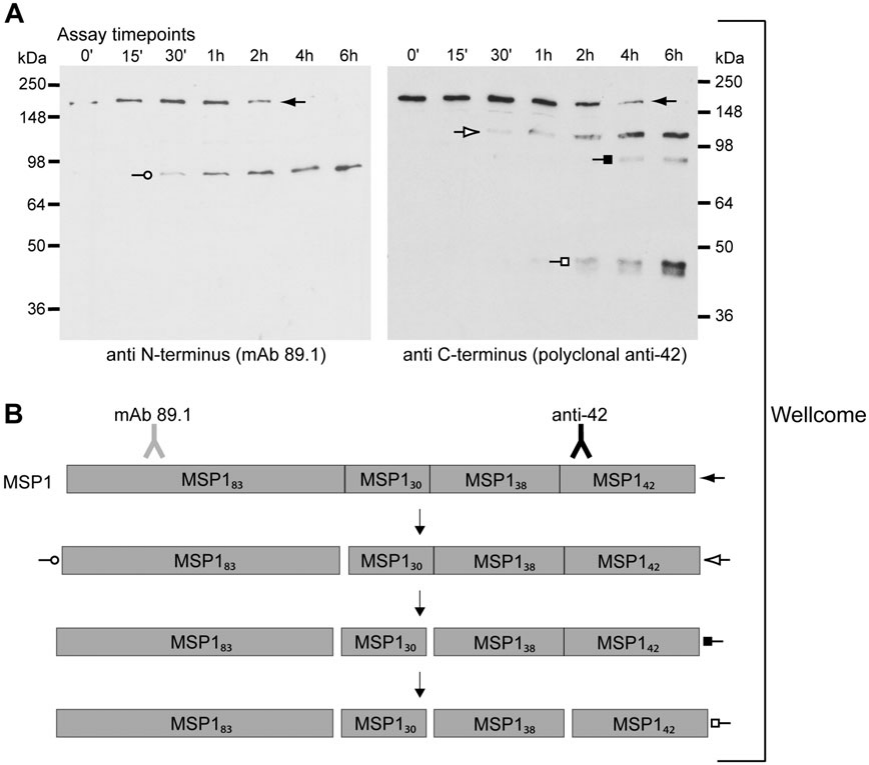
PfSUB1-mediated proteolytic processing of the Wellcome-type MSP1 is ordered.A. Typical time-course of digestion of native T9/94 *P. falciparum*-derived MSP1. Saponin-permeabilized T9/94 schizonts were incubated at 37°C in the presence of rPfSUB1. Samples taken at intervals were analysed by Western blot, probing with either the MSP1_83_-specific mAb 89.1 (left-hand side), or a MSP1_42_-specific polyclonal antibody (right-hand side). For both blots the MSP1 precursor is indicated with a black arrow, processing intermediates are marked with an open arrow, or closed square-head lollipop, while terminal processing products are marked with an open round or square-head lollipop. No processing was observed in the absence of added rPfSUB1 (not shown).B. Schematic of the order of Wellcome-type MSP1 primary processing, determined from the digestion assay shown in A. Individual processed species are marked with arrow or lollipop symbols to aid comparison with the corresponding bands on the blots.

To explore whether the above-described processing pattern holds for the other major dimorphic form of MSP1, we returned to the cell-based processing assay, this time using permeabilized schizonts from a parasite clone (T9/94) expressing the Wellcome-type MSP1. In this case formation of MSP1_83_ did not proceed through an intermediate detectable with mAb 89.1 (Fig. 3A), suggesting rapid, preferred cleavage at the 83/30 site. In contrast, the antibodies reactive with the C-terminal MSP1_42_ fragment detected two transient intermediates much larger than the final MSP1_42_ product, suggesting that cleavage at both the 83/30 and 30/38 sites took place more rapidly than cleavage at the 38/42 position (Fig. 3B). Collectively, our results suggest that for both major forms of MSP1, primary processing by PfSUB1 is an ordered event. This order appears different between the MSP1 types, but in both cases cleavage at the 38/42 site occurs last.

### Peptides based on the 38/42 MSP1 processing site are poor PfSUB1 substrates relative to peptides based on the other primary processing sites

**Fig. 4:**
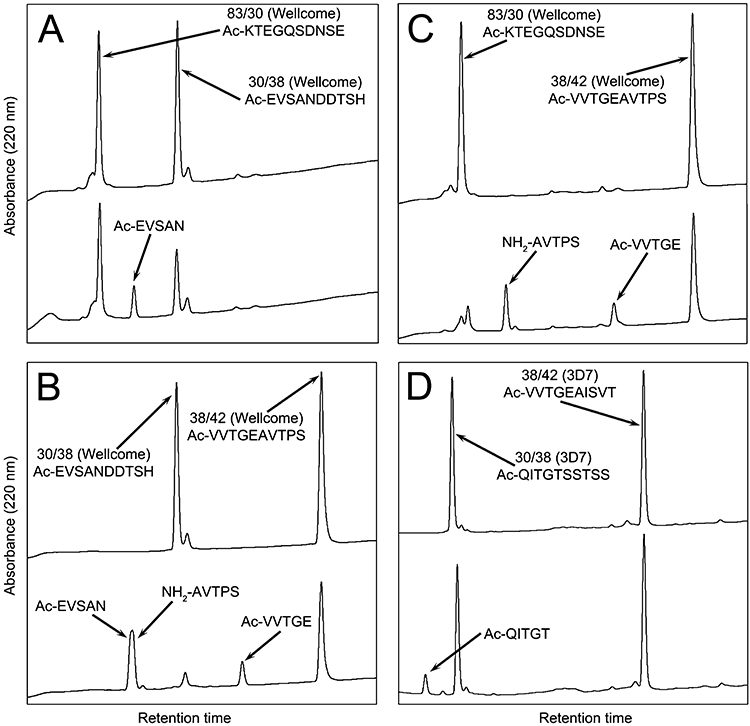
Synthetic peptides based on the 38/42 processing sites in the 3D7 and Wellcome-type MSP1 are poor substrates for PfSUB1.RP-HPLC chromatograms showing partial digestion by rPfSUB1 of N-acetylated decapeptides based on the primary processing sites in the Wellcome-type (A–C) and 3D7-type (D) MSP1. The top chromatogram in each panel shows the elution profile of an equimolar mix of the undigested peptides, while the lower chromatogram shows the RP-HPLC elution profile after partial digestion with rPfSUB1. Identities of major peaks as determined by electrospray mass spectrometry (data not shown but see Koussis *et al.*, 2009), are indicated. As described previously (Yeoh *et al.*, 2007; Koussis *et al.*, 2009), highly polar cleavage products were usually not retained by the RP-HPLC column but eluted in the column flow-through. Note that for determination of relative initial rates of cleavage, cleavage rates were compared when the faster-cleaved peptide of each pair was digested by ≤ 10%, as described in *Experimental procedures*. For clarity, digestion was allowed to proceed much more than 10% in most of the profiles shown in this figure.

Previous work has shown that short synthetic peptides based on known PfSUB1 cleavage sites – including the *P. falciparum* MSP1 primary processing sites – can act as substrates for the protease and are correctly cleaved by rPfSUB1 (Yeoh *et al.*, 2007; Koussis *et al.*, 2009). To investigate whether the ordered primary processing of MSP1 by PfSUB1 was a result of the differences in primary amino acid sequence at the various sites, peptides based on the three positionally conserved processing sites (the 83/30, 30/38 and 38/42 sites) were compared for their susceptibility to cleavage by PfSUB1. To do this, pairs of N-acetylated decapeptides corresponding to sequences encompassing the sites were mixed at equimolar ratios, then initial rates of cleavage by rPfSUB1 established by analytical reversed-phase high-performance liquid chromatography (RP-HPLC). Under such conditions, because the peptides effectively act as competing substrates, their relative initial rates of hydrolysis are a function of the ability of PfSUB1 to discriminate between them, and so are directly proportional to their relative *k*_cat_/*K*_m_ values, irrespective of their actual concentration (Cornish-Bowden, 2004). This analysis allowed us to estimate relative initial cleavage rates between peptide pairs, and to confidently rank most of the peptides in order of their susceptibility to cleavage. For the three Wellcome MSP1-derived peptides (Fig. 4A–C), the rank order was 30/38 > 83/30 > 38/42 in order of best to worst substrate, with the 30/38 peptide being initially cleaved approximately twofold more rapidly than the 83/30 peptide, and the 83/30 cleaved fivefold more rapidly than the 38/42 peptide. This order is different to that observed when using the intact protein as substrate, but in both cases the 38/42 site appeared to be the least preferred. In the case of the 3D7 MSP1-derived peptides, the peptide based on the 83/30 site behaved aberrantly under the RP-HPLC conditions used, eluting as a very broad peak, and so was excluded from the comparison. However, a comparison of the 3D7-type 30/38 and 38/42 peptides (Fig. 4D) showed again that the 30/38 peptide was cleaved much faster (initial rate 17-fold faster) than the 38/42 peptide. These results show that peptides based on the 38/42 sites of both the Wellcome and 3D7 MSP1 are very poor substrates compared with all other peptides tested. This is consistent with our analysis of cleavage of intact MSP1, and suggests that the reason why cleavage at the 38/42 sites occurs more slowly than cleavage at the other primary processing sites is at least in part due to the less-preferred nature of the primary sequence at those sites.

### Substitution of the MSP1 secondary processing site with a PfSUB1-sensitive site alters the pattern of primary processing and cannot be tolerated by the parasite

**Fig. 5:**
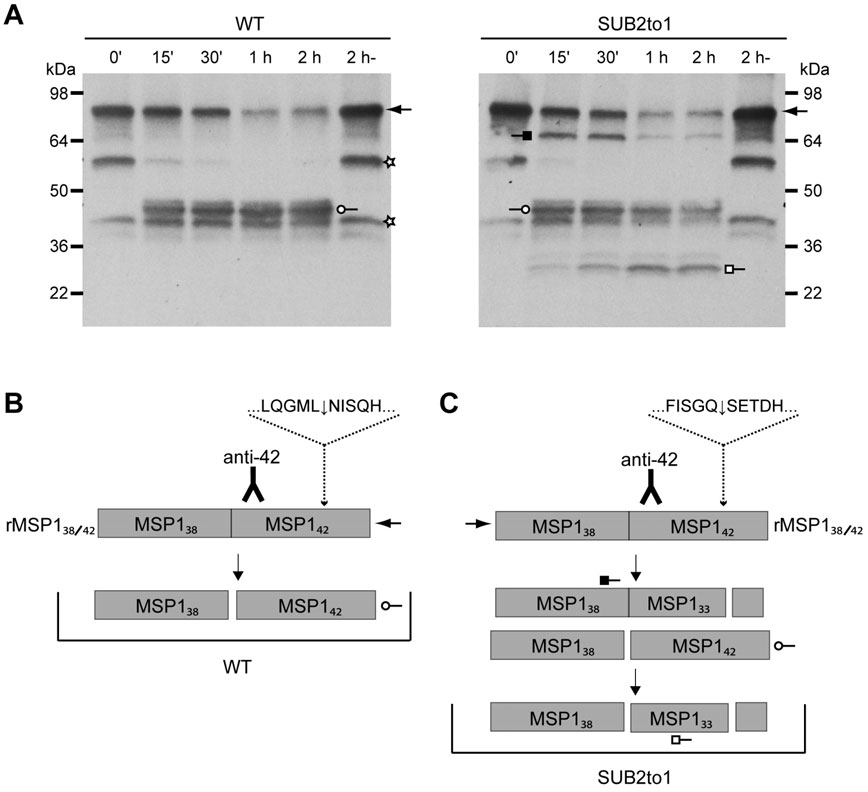
Substitution of the MSP1 secondary processing site with a PfSUB1-sensitive sequence.A. Western blot time-course analysis of rPfSUB1-mediated processing of rMSP1_38/42_ either in wild-type form (WT) or after modification of the secondary processing site by site-directed mutagenesis to convert it to a predicted PfSUB1-sensitive site (SUB2to1). The WT rMSP1_38/42_ (80 kDa, arrowed) is converted to a stable 42 kDa terminal species (open round-head lollipop) equivalent to MSP1_42_. The SUB2to1 mutant (arrowed) is similarly converted to the normal MSP1_42_ species (open round-head lollipop), but also undergoes an additional processing step that converts it via an approximate 70 kDa intermediate (closed square-head lollipop) to a stable 33 kDa form (open square-head lollipop). The 2 h tracks show the results of incubation for 2 h in the absence of added rPfSUB1. The bands marked with a star are likely truncated forms of rMSP1_38/42_ derived from breakdown of the recombinant protein during its purification and refolding.B and C. Schematic depiction of processing patterns for the WT (B) and SUB2to1 mutant rMSP1_38/42_ protein (C). The relative position of the secondary processing site, the sequence modifications made and the region of the protein recognized by the rabbit anti-MSP1_42_ antibodies used to probe the blots, are indicated. Individual processed species are marked with arrow or lollipop symbols to aid comparison with the corresponding bands on the blots.

Following primary processing by PfSUB1 and merozoite egress, the entire MSP1/6/7 complex is shed from the free merozoite surface by a single further cleavage at a QG/DML↓NISQ motif, mediated by PfSUB2 (Fig. 1). This secondary processing site is completely refractory to PfSUB1 (Koussis *et al.*, 2009). To assess the biological importance of MSP1 processing, we next investigated the effect on parasite viability of selectively perturbing the processing. To do this, we decided to examine whether the secondary processing site could be modified to render it cleavable by PfSUB1, anticipating that this might result in cleavage at the secondary processing occurring prematurely, concomitant with primary processing. As the time-scale between discharge of PfSUB1 into the PV and eventual schizont rupture may be very short (minutes or even seconds), we considered it important that – to guarantee cleavage at the modified site – we inserted a site that was more efficiently cleaved than the least-preferred primary processing site, the 38/42 site. Based on the PfSUB1 consensus recognition motif, described above, we designed an ‘artificial’ sequence, FISGQ↓SETDH (the arrow indicates the predicted scissile bond), which we predicted would be efficiently cleaved by PfSUB1. To confirm this, we first tested the synthetic decapeptide Ac-FISGQSETDH for sensitivity to cleavage *in vitro* by rPfSUB1. As shown in Fig. S2, this peptide was indeed cleaved at the predicted Gln–Ser bond, with substantially faster kinetics (approximately 4.5-fold faster) than both the 3D7 and Wellcome-type 38/42 peptides. To test the amino acid sequence for susceptibility to PfSUB1 in the context of an intact protein, we then introduced the same sequence into a recombinant form of the C-terminal ‘half’ of the Wellcome MSP1 (called rMSP1_38/42_), using site-directed mutagenesis to replace residues flanking the secondary processing site. As shown in Fig. 5, incubation of this modified recombinant protein with rPfSUB1 resulted in an additional specific processing step, consistent with cleavage at the modified site and resulting in the formation of a 33 kDa processing product analogous to that produced upon PfSUB2-mediated secondary processing of the authentic parasite MSP1 (Blackman and Holder, 1992). Conversion to the 33 kDa product took place with production of an intermediate species likely resulting from cleavage at the introduced site before cleavage at the 38/42 site (Fig. 5A and C). These results provided convincing evidence to suggest that, when introduced into MSP1, the artificial sequence could be recognized and cleaved by PfSUB1 in a similar manner to an authentic PfSUB1 cleavage site. Importantly, the indications that it was a better substrate than the 38/42 sequence encouraged us in our prediction that it would be efficiently cleaved by PfSUB1 when incorporated into the authentic, parasite-surface MSP1.

**Fig. 6:**
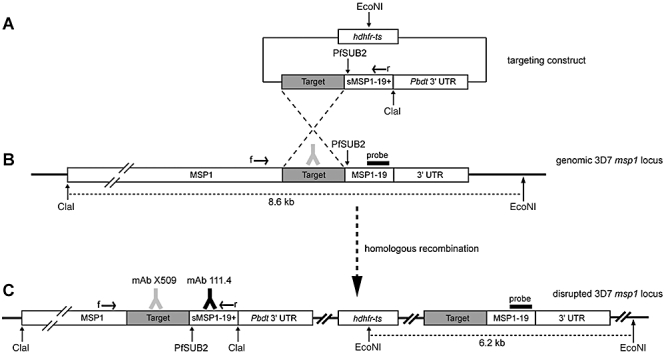
Strategy for modification of the MSP1 secondary processing site in *P. falciparum* by single cross-over homologous recombination.A. Integration construct (pMSP1chimWT or pMSP1chimSUB2to1) used for targeted homologous recombination. The plasmid contains 1036 bp of targeting sequence (dark grey) derived from the 3D7 *msp1* gene, fused in frame to synthetic recodonized sequence (sMSP1-19+) encoding the C-terminal 119 residues of the Wellcome-type MSP1, including its GPI anchor sequence. The position of the PfSUB2 cleavage (the secondary processing site) is arrowed. In pMSP1chimSUB2to1 this site is modified to encode a PfSUB1-sensitive sequence, but otherwise the two constructs are identical. Because the recodonized sequence shares low identity with the authentic *msp1* sequence, cross-over (dotted lines) is expected to occur only within the targeting sequence. *Pbdt* 3′ untranslated region (UTR), UTR of the *Plasmodium berghei* dihydrofolate reductase gene to ensure correct transcription termination and polyadenylation of the modified gene. *hdhfr-ts*, human dihydrofolate reductase–thymidylate synthase cassette, providing resistance to the antifolate drug WR99210.B. The 3D7 *P. falciparum* genomic *msp1* locus.C. Expected result of integration, which displaces the 3′ end of the *msp1* gene downstream, and replaces it with a chimera of the native sequence and the recodonized Wellcome-type sequence. As the integration plasmid contains only a partial *msp1* open reading frame, not preceded by a promoter, the only copy to be transcribed is the modified chimeric *msp1* gene directly downstream of the endogenous promoter. The positions of epitopes recognized by mAbs X509 and 111.4 are indicated. Restriction sites used for Southern blot analysis (see Fig. 8), the predicted sizes of restriction products, and the position of hybridization of the probe used for Southern analysis are shown.

In light of the above findings, we set about investigating the consequences of similarly modifying the *msp1* gene in the 3D7 *P. falciparum* clone, using targeted homologous recombination. For this we exploited a previously used strategy (Yeoh *et al.*, 2007) in which we introduced into the parasite circular DNA constructs containing targeting sequence corresponding to internal *msp1* sequence, fused in frame to a short stretch of synthetic ‘recodonized’ sequence encoding the C-terminal domain of MSP1 (Figs 6 and S3). The synthetic sequence shares low identity at the nucleotide level with the authentic *msp1* gene, reducing the likelihood of undesired recombination downstream of the junction between the authentic and synthetic sequence. Two integration constructs were used, one of which (pMSP1chimWT) was designed to leave the amino acid sequence at the secondary processing site unaltered following integration, while the other (pMSP1chimSUB2to1) was designed to replace the secondary processing site with the FISGQ↓SETDH sequence shown above to be efficiently targeted by PfSUB1. The DNA constructs were otherwise identical. In addition, the constructs were designed to produce upon integration a chimeric gene in which the 3′ region (the synthetic sequence) encoded a Wellcome-type MSP1 C-terminal domain. This differs from the 3D7 sequence at just 5-amino-acid residues. Importantly, it can be distinguished from the 3D7-type domain by its reactivity with mAb 111.4, the epitope of which is not present in the 3D7 MSP1 as it requires the presence of a Gln residue at a dimorphic position 14 residues downstream of the secondary processing site (Fig. S3) (Chappel and Holder, 1993; Tolle *et al.*, 1995); the 3D7 sequence has a Glu residue at that position. Thus, correct integration of either construct was expected to effectively epitope-tag the resulting MSP1 chimeric gene product, enabling confirmation of the desired homologous recombination event at the protein level.

**Fig. 8:**
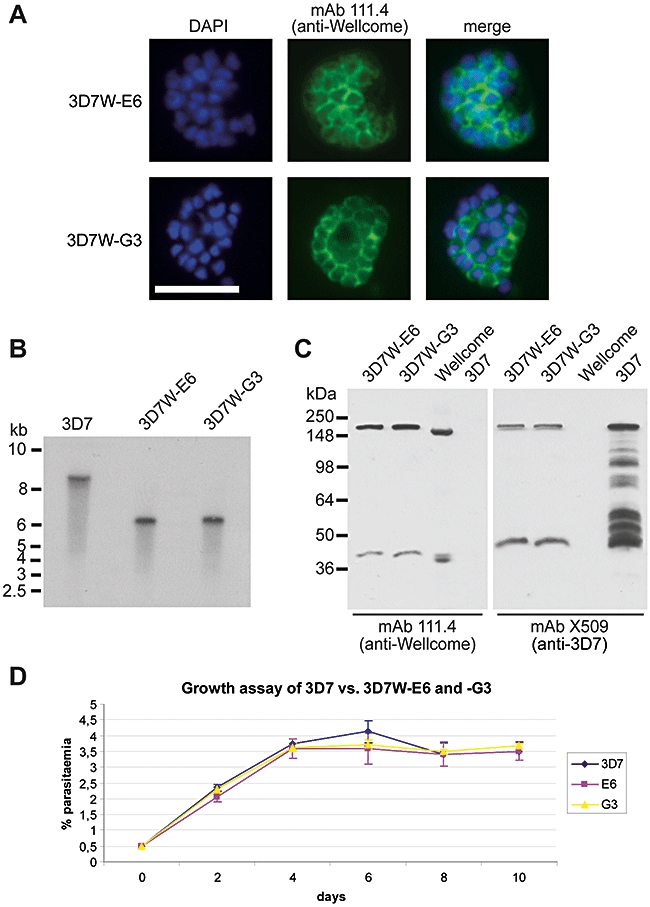
Transgenic *P. falciparum* clones stably expressing a chimeric MSP1 with an unmodified secondary processing site are phenotypically normal.A. IFA examination of transgenic clones 3D7W-E6 and 3D7W-G3 shows reactivity with the Wellcome-specific mAb 111.4, confirming stable expression of a chimeric form of MSP1. Nuclei were counterstained with DAPI. Scale bar, 5 µm.B. Southern blot analysis. Genomic DNA from parental wild-type 3D7 parasites, and clones 3D7W-E6 and 3D7W-G3, was digested with ClaI and EcoNI and analysed by Southern blot using a 480 bp PCR product amplified from the 3′ region of the 3D7 *msp1* gene (see Fig. 6). The endogenous locus band is visible at 8.6 kb in the parental 3D7 digest, whereas its replacement with a 6.2 kb signal in the clone DNA digests is consistent with modification of the *msp1* locus as predicted (see Fig. 6).C. Western blot confirms expression of chimeric MSP1 in the transgenic clones. Schizont extracts from clones 3D7W-E6 and 3D7W-G3, as well as from parental 3D7 and T9/94 (Wellcome-type) parasites, were fractionated on 10% SDS-PAGE gels and probed with either mAb 111.4 or mAb X509. In contrast to the parental extracts, the full-length MSP1 and MSP1_42_ species from the transgenic clones were recognized by both mAbs. Note that mAb 111.4 recognizes a reduction-sensitive epitope, so the left-hand blot was from a gel run under non-reducing conditions, whereas the right-hand blot was from a gel run under reducing conditions.D. Growth assay comparing *in vitro* replication rates of parental 3D7 and transgenic clones 3D7W-E6 and 3D7W-G3. All three clones were co-synchronized to a 2 h window following 8 days (four intraerythrocytic cycles) of culture in the absence of drug pressure). Initial parasitaemias, measured by FACS, were adjusted to 0.5%, then subsequently assessed at 48 h intervals and cultures diluted as described in *Experimental procedures*. Individual points represent mean values from triplicate samples for all three lines and error bars represent the standard deviations of these values. There was no significant difference in growth rates of the clones.

**Fig. 7:**
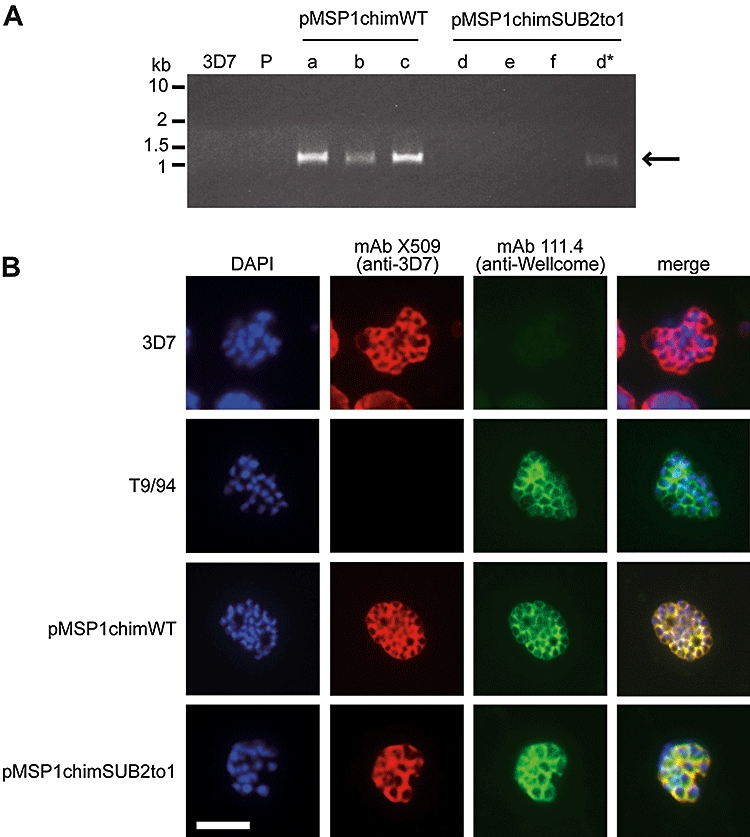
Production of transgenic *P. falciparum* lines expressing chimeric MSP1.A. PCR analysis to detect correct genomic integration of the homologous recombination constructs pMSP1chimWT and pMSP1chimSUB2to1. As controls, template DNA was prepared from the parental 3D7 clone, or input pMSP1chimWT plasmid DNA only (P). For test samples, genomic DNA was prepared after four rounds of drug cycling from three lines transfected with pMSP1chimWT (a–c), or three lines transfected with pMSP1chimSUB2to1 (d–f), or from one of the pMSP1chimSUB2to1-transfected line following seven drug cycles (d*). Oligonucleotide primers used in the PCR were designed to hybridize upstream of the region used as the targeting sequence in the 3D7 MSP1 locus, as well as to an allele-specific portion of the recodonized Wellcome sMSP1-19+ sequence in the transfection constructs (see Fig. 6C). A PCR product of approximately 1.2 kb can therefore only be produced if the predicted integration event has taken place. The integration-specific PCR product was detected for all three pMSP1chimWT-transfected lines but not for the pMSP1chimSUB2to1-transfected lines, except for one line examined after seven drug cycles (d*) (arrowed).B. IFA examination of the uncloned transgenic lines confirms expression of chimeric forms of MSP1. Samples of the pMSP1chimWT-transfected line (line a, drug cycle 4) and the pMSP1chimSUB2to1-transfected line (line d*, drug cycle 8) were double-probed with the 3D7 MSP1-specific mAb X509 and the Wellcome MSP1-specific mAb 111.4. Whereas the parental 3D7 and T9/94 parasite lines showed reactivity only with one or other type-specific mAb as expected, both transgenic populations contained parasites reactive with both mAbs, indicating expression of the predicted chimeric MSP1. The proportion of schizonts in the transgenic lines reactive with both antibodies was approximately 95% for the pMSP1chimWT-transfected line, and approximately 0.2% for the pMSP1chimSUB2to1-transfected line. Nuclei were counterstained with DAPI. Scale bar, 5 µm.

The pMSP1chimWT and pMSP1chimSUB2to1 constructs were separately transfected in triplicate into 3D7 parasites and the cultures drug cycled to select for integration events. After four cycles of drug treatment, polymerase chain reaction (PCR) analysis indicated the presence of homologous recombination events in all three parasite lines transfected with pMSP1chimWT (Fig. 7A). In contrast, a weak PCR-positive result was obtained with only one of the lines transfected with pMSP1chimSUB2to1, and then only after seven drug cycles over an extended period of 8 months (Fig. 7A), suggesting that integration of that construct into the parasite genome was not favoured. Sequencing of all four integration-specific PCR products confirmed that homologous recombination had occurred, with cross-over taking place upstream of the synthetic recodonized Wellcome sequence, and that for the line transfected with pMSP1chimSUB2to1, the SUB2to1 mutation had been incorporated into the 3D7 *msp1* locus as expected (data not shown). To confirm expression of the expected chimeric MSP1 gene product in the transgenic lines, all four lines were analysed by immunofluorescence assay (IFA) using both mAb X509 (which recognizes a 3D7-specific epitope within the N-terminal region of MSP1_42_), and the Wellcome type-specific mAb 111.4. As expected, a significant proportion (approximately 95%) of schizonts in the lines transfected with pMSP1chimWT showed reactivity with both mAbs (Fig. 7B), confirming that they expressed a chimeric MSP1 distinct from the parental 3D7 MSP1. In contrast, only approximately 0.2% of schizonts in the line transfected with pMSP1chimSUB2to1 showed reactivity with both antibodies. The pattern of reactivity was identical to that in the pMSP1chimWT line, indicating correct expression and subcellular localization of the chimeric mutant MSP1. However, the low proportion of parasites expressing the mutant protein could not be increased by further drug cycling, and in fact these presumed transgenic parasites could only be maintained in the population by continued drug cycling.

One of the pMSP1chimWT-transfected parasite lines (at drug cycle 2) and the PCR-positive pMSP1chimSUB2to1-transfected line (at drug cycle 8) were cloned by limiting dilution, and individual clones were again examined by IFA. No mAb 111.4-positive clones were obtained from the pMSP1chimSUB2to1-transfected line, despite extensive drug cycling and four independent cloning attempts. In contrast, mAb 111.4-positive clones were easily obtained from the pMSP1chimWT-transfected parasite line. Two of these, clones 3D7W-E6 and 3D7W-G3, were selected and examined further by PCR (not shown) and Southern blot. This confirmed the expected genomic rearrangements at the *msp1* locus (Fig. 8B). In further support of this, the MSP1 protein expressed by the two clones was reactive in Western blots with both the 3D7-specific mAb X509 and the Wellcome-specific mAb 111.4, confirming its chimeric nature (Fig. 8C). The clones were maintained routinely under drug pressure (2.5 nM WR9921) to select against any possibility of reversion to wild type by resolution of the single cross-over event used to produce them. However, the clones remained reactive with mAb 111.4 even after prolonged culture (> eight intraerythrocytic cycles) in the absence of drug (not shown). To assess the impact of the MSP1 modification on parasite fitness, the transgenic clones were compared with the parental 3D7 clone in growth rate assays. No differences in growth rates were detected (Fig. 8D), showing that expression of the chimeric MSP1 containing an authentic PfSUB2 cleavage site has no discernible effect on parasite viability. Collectively, the ease with which these transgenic parasites were established, compared with our inability to isolate similar transgenic parasites in which the secondary processing site in MSP1 was replaced with a PfSUB1-sensitive site strongly suggests that interfering with the regulation of MSP1 processing has a substantial deleterious impact on parasite viability.

## Discussion

MSP1 has attracted widespread interest in the malaria research field over the last three decades. A steady accumulation of evidence that the protein plays a key role in erythrocyte invasion by the malaria merozoite, bolstered by extensive data supporting its potential as a candidate vaccine antigen, have prompted efforts to understand the function of MSP1. The fact that MSP1 is dramatically proteolytically modified at or around the point of invasion has also stimulated much investigation. We have now exploited knowledge of the substrate specificity of the proteases involved, PfSUB1 and PfSUB2, to obtain the first genetic evidence that proteolytic maturation of MSP1 is critical for parasite viability, and therefore – by implication – for the function of MSP1.

Ordered proteolytic processing is a feature of several systems in which limited proteolysis induces structural changes in proteins required for their functional maturation. Particularly well-studied examples of this include the protease-mediated structural reorganization of viral capsid proteins necessary for their conversion into mature, infectious forms [for reviews see Steven *et al.* (2005); Byrd and Hruby (2006); Ganser-Pornillos *et al.* (2008)]. In the case of retroviral Gag polyprotein, which is cleaved at several positions by the same virally encoded aspartic protease, mutations that prevent cleavage at certain sites, or that induce imprecise cleavage, or that alter the order of cleavage, all severely ablate viral infectivity (e.g. Craven *et al.*, 1993; Pettit *et al.*, 1994). These and other similar observations have led to suggestions that ordered cleavage has an important role in assuring the irreversibility of the induced conformational shifts that lead to the formation of the mature virus particle. Our examination of rPfSUB1-mediated cleavage of both parasite-derived and recombinant MSP1 indicated a hierarchy of cleavage rates at the different primary processing sites which – though slightly different between the two major MSP1 types – in both cases indicated that cleavage at the 38/42 site occurred last. Simplistically, ordered cleavage at multiple sites by a single protease may be predicted to be determined by either the primary amino acid sequence at the processing sites or the accessibility of the various sites to the protease. Should cleavage induce conformational changes in a protein, the latter element might alter during the course of processing, and therefore cleavage at particular sites may depend on prior processing at other sites. Our further examination of MSP1 primary processing using peptides based on the different sites was therefore important in that it suggested that the order may at least in part be explained by the primary amino acid sequence at the different sites; steady-state cleavage rates of peptides based on the 38/42 sites were in all cases slower than that of the other peptides tested. The correlation between the protein and peptide data was not perfect, however; the observation that processing of the 83/30 site in the Wellcome-type MSP1 takes place before cleavage at the central 30/38 site was not consistent with the corresponding peptide results, where the 30/38 peptide was clearly cleaved more rapidly than the 83/30 peptide. We did not examine this issue further here, but it does suggest that factors other than primary sequence may be important in determining the order of cleavage. Whatever the case, by analogy with the examples referred to above, our finding that proteolytic processing of MSP1 by PfSUB1 is ordered is consistent with the proteolysis having a functional role. It may be important for the 38/42 site to be processed last, and the limited sequence divergence between the two major MSP1 forms at this site may indicate selective pressure to maintain this property. It is worth noting that cleavage by PfSUB1 of SERA5, a PV-resident putative papain-like protease, also occurs in a stepwise manner, with cleavage at a position referred to as site 1 preceding cleavage at a second sequence referred to as site 2 (Li *et al.*, 2002; Yeoh *et al.*, 2007). Peptides based on site 1 are much better substrates that those based on site 2 (Yeoh *et al.*, 2007), again consistent with the notion that the rate of cleavage by PfSUB1 is largely dependent on the primary sequence flanking the sites.

To evaluate the biological importance of the two stages of MSP1 processing, we next explored the effects of exchanging the secondary processing site, normally refractory to PfSUB1, with a sequence that is sensitive to that protease. We predicted this to have either or both of two possible consequences: it could result in premature ‘secondary processing’ as a result of cleavage by PfSUB1 at the modified site while the merozoite was still intracellular; and/or it could impact on PfSUB2 recognition of the site, inhibiting or otherwise modifying shedding of MSP1 following egress. We were unable to directly assess the likelihood of the second possibility, as we do not have access to recombinant PfSUB2. However, the apparent sequence-independent nature of PfSUB2 cleavage (Howell *et al.*, 2003; Harris *et al.*, 2005) leads us to favour the first possibility. In support of that we found that the peptide Ac-FISGQSETDH was a better substrate for PfSUB1 than either of the peptides based on the 38/42 sites, and that introduction of the FISGQSETDH sequence into a recombinant protein corresponding to the C-terminal ‘half’ of MSP1 rendered the protein susceptible to an additional cleavage on exposure to rPfSUB1 *in vitro*, with a pattern consistent with efficient cleavage at the modified secondary processing site. Introduction of this sequence into the endogenous 3D7 parasite *msp1* gene was achieved by targeted homologous recombination, as convincingly demonstrated by both PCR and IFA, and eventually resulted in the establishment of viable transgenic parasites harbouring the mutation. However, they grew poorly and, despite our best efforts, could not be cloned or grown to a sufficiently high parasitaemia to enable biochemical analysis. This precluded a detailed characterization of the phenotype. However, we believe that the most likely explanation for the deleterious effects of the introduced mutation was that it resulted in premature shedding of MSP1 as a result of PfSUB1 cleavage at the modified secondary processing site following discharge of the protease into the PV just before egress.

The function of MSP1 primary processing is unclear. However, there is evidence that it may impact on its capacity to interact with key partner proteins, as well as on its putative role as an adhesin during invasion. In an investigation of the MSP1/6/7 complex, Kauth *et al.* (2006) found that a recombinant form of MSP6 could interact with recombinant MSP1_38_, but not with full-length MSP1, suggesting that primary processing is required for MSP6 binding. More recently, Boyle *et al.* (2010) demonstrated that the MSP1_42_ processing product – but not full-length MSP1 – binds to heparin and similar sulphated polysaccharides, suggesting a role for the processed protein in adhesive interactions with erythrocyte surface proteoglycans. Both these findings are consistent with the notion that primary processing functionally activates MSP1, and they will provide valuable surrogate markers for future work investigating the functional consequences of blocking MSP1 cleavage at specific primary processing sites.

In conclusion, our findings implicate proteolytic processing of MSP1 as a highly regulated process that is important for viability of the asexual blood-stage malaria parasite. Furthermore, we have established a link between the ordered proteolytic maturation of this key merozoite surface component and its function. This emphasizes the potential of the two enzymes involved – PfSUB1 and PfSUB2 – as targets for antimalarial drug development. Finally, our observations add further support to the notion that the PV has a dual role: it serves not only as a protective intracellular environment within which the parasite replicates sequestered from the host immune system, but also functions as a regulated processing compartment in which daughter merozoites undergo maturation to their full invasive capacity before release into the bloodstream.

## Experimental procedures

### Bioinformatic analysis

All the *P. falciparum* MSP1 primary processing sites have been precisely mapped [see Koussis *et al.* (2009) and references contained therein]. For multiple alignment of regions around these sites, sequences were taken from *P. falciparum* MSP1 sequences deposited in PlasmoDB (http://plasmodb.org/plasmo/) and GenBank (http://www.ncbi.nlm.nih.gov/). The sequences were also examined in comparison with all deposited MSP1 sequences using WuBlast (http://www.ebi.ac.uk/Tools/blast2/index.html) and aligned using Clustalw. In each case, in addition to the 10 residues (P5–P5′) flanking the cleavage sites, a further 20 amino acids extending beyond the P5 and P5′ positions were included in the analysis.

### Parasite maintenance, transfection and growth assays

Maintenance and synchronization *in vitro* of asexual blood stages of *P. falciparum* clones 3D7 and T9/94, and purification of schizonts was as previously described (Blackman *et al.*, 1994; Yeoh *et al.*, 2007). For determination of parasitaemia by flow cytometry, parasite DNA was stained with hydroethidine (Bergmann-Leitner *et al.*, 2006). Fifty microlitres of culture was diluted into 500 µl of PBS in FACS tubes. Hydroethidine was added directly to the dilute culture to a final concentration of 50 µg ml^−1^. The samples were then incubated in the dark at 37°C for 20 min, then 1 ml of PBS was then added to each sample to reduce further staining and the samples kept on ice in the dark for up to 2 h. Samples were then analysed on a BD FACSCalibur machine, using BD CellQuest™ Pro V5.2 software. Forward scatter/side scatter (FS/SS) was adjusted to gate for all cells (G1). Approximate values FS = E – 1 log, SS = 258 log. Fluorescence detector 2 was set to 309–330 mV, log scale. The red fluorescent population was gated as G2. One hundred thousand events were counted for the entire sample within G1, with G2 counting the proportion of the 100 000 cells that were fluorescent.

For transfection, ring-stage parasites were electroporated with plasmid DNA as described previously (Harris *et al.*, 2005). After initial selection using 2.5 nM WR99210 (Jacobus Pharmaceuticals, NJ, USA) for approximately 4 weeks, parasites were subjected to repeated cycles of drug selection for 3 weeks followed by removal of the drug for 3 weeks (‘drug cycling’). Clonal populations were obtained by limiting dilution.

For growth assays, parental and transgenic parasite clones were tightly synchronized to a 2 h window. Twenty-four hours later (at mid trophozoite stage), all cultures were adjusted to 0.5% parasitaemia and 2% haematocrit, then triplicate cultures of each clone were set up in 25 cm flasks and parasitaemia assessed by FACS at 48 h intervals for the ensuing 10 days (five cycles). Following each count, the dilution factor required to adjust the 3D7 control culture back down to a parasitaemia of approximately 4% was calculated. All the lines were then all diluted by this same factor, using fresh erythrocytes in medium at a 2% haematocrit.

### MSP1 processing assays

For examination of MSP1 proteolytic processing by PfSUB1, samples of recombinant MSP1 or parasite-derived native MSP1 were digested for periods of up to 24 h in an adaptation of a previously described method (Koussis *et al.*, 2009). Briefly, either 200 µg of full-length recombinant 3D7 MSP1, or protease inhibitor-treated and saponin-permeabilized 36- to 42-h-old 3D7 or T9/94 schizonts in PfSUB1 digestion buffer (25 mM HEPES pH 7.4, 12 mM CaCl_2_) were supplemented with purified recombinant PfSUB1 (rPfSUB1) and incubated at 37°C. Samples taken at intervals were immediately solubilized with SDS-PAGE sample buffer containing 50 mM DTT. Upon completion of the assay, all samples were subjected to SDS-PAGE and analysed by Western blot.

### Peptides and RP-HPLC

All synthetic peptides used were synthesized by standard solid-phase Fmoc chemistry either in-house at NIMR or by BIOMATIK Corporation (http://www.biomatik.com/). Peptide Ac-CQDMLNISQHC based on the PfSUB2 secondary processing site in MSP1 was synthesized for a previous study (P.K. Harris, PhD thesis). Preparation of stock and working solutions of peptides, analysis of peptide cleavage by RP-HPLC and mass spectrometric identification of digestion products, was as previously described (Koussis *et al.*, 2009). For quantitative determination of relative initial rates of cleavage of equimolar mixtures of peptides, cleavage rates were compared after no more than 10% of the fastest cleaved peptide had been hydrolysed, as described previously (Withers-Martinez *et al.*, 2002).

### Expression, purification and PfSUB1 digestion of recombinant Wellcome MSP1_38/42_

The *Escherichia coli* expression vector pZE23-f38/42, encoding a recombinant form of the C-terminal ‘half’ of the Wellcome-type MSP1 (termed rMSP1_38/42_) was modified by site-directed mutagenesis to replace sequence encoding the PfSUB2 cleavage site QGML↓NISQ with an artificial predicted PfSUB1 cleavage motif, ISGQ↓SETD, creating plasmid pZE23-f38/42-SUB2to1. Both wild-type and SUB2to1 mutant vectors were introduced into *E. coli* strain BL21 Gold. Induction of expression, purification of recombinant protein from inclusion bodies and refolding of the protein was as described previously (Epp *et al.*, 2003). Refolded protein was rapidly diluted into PfSUB1 digestion buffer and used immediately.

### Construction of integration constructs pMSP1chimWT and pMSP1chimSUB2to1

A synthetic gene fragment codon-optimized for expression in *E. coli* and encoding the C-terminal region of the Wellcome-type MSP1 (amino acid residues 1521–1639) was synthesized by Geneart AG and provided as an insert with terminal BglII and XhoI sites in pGA4 (ampR). The synthetic gene fragment was excised with BglII and XhoI, and subcloned into identical sites in the construct pHH1–ΔSERA4 (a kind gift of Dr Brendan Crabb, Burnet Institute, Melbourne, Australia) to create pHH1–ga–MSP1. A 1036 bp upstream region of the 3D7 *msp1* coding sequence was amplified by PCR from 3D7 genomic DNA using forward and reverse primers designed to incorporate 5′ BclI, and 3′ PstI restriction enzyme sites. The PCR product was digested with BclI and PstI, and subcloned into pHH1–ga–MSP1 between the BglII and PstI sites to create pMSP1chimWT. Sequence encoding the PfSUB2 processing site (QGML↓NISQ) in the construct was mutated to the predicted PfSUB1 cleavage site ISGQ↓SETD by site-directed mutagenesis using a QuikChange II XL kit (Qiagen). Sequence flanking this modified fragment was then excised with BglII and ClaI and subcloned between identical sites in pMSP1chimWT to create pMSP1chimSUB2to1. Nucleotide sequences of all cloned products were confirmed by sequencing on both strands.

### Southern blot and PCR analysis of transgenic parasite lines

Parasite genomic DNA was prepared as previously described (Harris *et al.*, 2005). For Southern blot analysis, DNA was digested with ClaI and EcoNI (Roche), electrophoresed on a 0.7% agarose gel and transferred to Hybond N Nylon membrane (Amersham Biosciences, Buckinghamshire, UK). Blots were probed with a [^32^P]-labelled, gel-purified, 480 bp PCR product amplified from the 3′ region of the 3D7 *msp1* gene. All oligonucleotide primers were obtained from Eurogentec. Stock solutions (100 µM in water) were stored at −80°C, and were used for PCR at a final concentration of either 5 or 10 µM. Primers designed to detect integration of transfected plasmids by PCR were kiMSP1pcrSC-FOR (5′-CCA AGT GAA AAT AAT AAG AAA GTT AAC G-3′; designed to hybridize to 3D7 *msp1* sequence upstream of the target sequence) and MSP1intmut-REV (5′-CCA GAT GAC GAA AGC AGC CGC TGT TCT GC-3′; designed to hybridize specifically with recodonized sequence encoding the C-terminal portion of the Wellcome-type *msp1* gene locus). The size of the expected PCR product, which can only be produced if integration has correctly taken place, is 1220 bp.

### Western blots and antibodies

Western blots were carried out as previously described (Jean *et al.*, 2003), probing with mAb 89.1 to detect MSP1_83_ (Holder *et al.*, 1985), mAb X509 or mAb 111.4 to detect 3D7- or Wellcome-type MSP1_42_ respectively (Holder *et al.*, 1985; Blackman *et al.*, 1991; Chappel and Holder, 1993; Tolle *et al.*, 1995), or rabbit antibodies specific for the Wellcome-type MSP1_42_ (Blackman *et al.*, 1993).

### IFA

Thin blood films containing *P. falciparum* schizonts were air-dried, fixed in 4% (w/v) paraformaldehyde for 30 min, permeabilized in 0.1% (w/v) Triton X-100 for 10 min, washed twice with PBS for 5 min and then blocked overnight at 4°C in 3% (w/v) BSA in PBS. Samples were probed with mAb 111.4 for 30 min at 37°C, then washed twice for 5 min in PBS. Secondary antibody (Alexa Fluor 594-conjugated anti-mouse IgG) was then applied for 30 min at 37°C. For double-staining this process was then repeated with the second antibody (mAb X509) followed by Alex Fluor 488-conjugated anti-human IgG. Samples were washed, stained with 4,6-diamidino-2-phenylindole (DAPI) for nuclear staining, then mounted in Citifluor (Citifluor, UK). Images were acquired using a Zeiss Axioplan 2 Imaging system (Carl Zeiss, Germany) and AxioVision 3.1 software. To estimate the proportion of schizonts in transgenic lines expressing chimeric forms of MSP1, schizonts were doubly probed probed with the 3D7 type-specific mAb X509 and the Wellcome type-specific mAb 111.4, then the proportion of 111.4-positive schizonts in a total of 2500 X509-positive schizonts estimated microscopically.
