# Supplementary material for: Regulated maturation of malaria merozoite surface protein-1 is essential for parasite growth
Source: Mol Microbiol. 2010 Aug 24;78(1):187–202. doi: 10.1111/j.1365-2958.2010.07324.x (PMC2995310; doi:10.1111/j.1365-2958.2010.07324.x)
Supplement: Supplementary file 1 [file mmi0078-0187-SD1.pdf]

Supplementary information

Regulated maturation of malaria merozoite surface protein-1 is essential for parasite growth

Matthew A Child[1], Christian Epp[2], Hermann Bujard[3] and Michael J Blackman[1*]

[1]Division of Parasitology, MRC National Institute for Medical Research, Mill Hill, London NW7 1AA, UK

[2]Department für Infektiologie-Parasitologie, Universitaetsklinikum Heidelberg, Im Neuenheimer Feld 324, D-69120 Heidelberg, Germany

[3]Zentrum für Molekulare Biologie Heidelberg (ZMBH), Universitaet Heidelberg, Im Neuenheimer Feld 282, D-69120 Heidelberg, Germany

* Correspondence: mblackm@nimr.mrc.ac.uk

Running title: MSP1 processing and malaria parasite viability.

Figure S1. PfSUB1-mediated proteolytic processing of recombinant 3D7 MSP1 is ordered. (**A**) Typical time course of digestion of full-length recombinant 3D7 *P. falciparum* MSP1. Recombinant protein (which possesses an N-terminal glutathione S-transferase tag, GST) was incubated at 37°C in the presence of rPfSUB1. Samples taken at intervals were analyzed by Western blot, probing with either the $MSP1_{83}$-specific mAb 89.1 (left hand side), or the $MSP1_{42}$-specific mAb X509 (right hand side). For both blots the MSP1 precursor is arrowed, processing intermediates are marked with a black round head lollipop, and terminal processing products are marked with a white square-head lollipop. No processing was observed in the absence of added rPfSUB1 (not shown). (**B**) Schematic depiction of the order of 3D7-type MSP1 primary processing as determined from the digestion assay shown in (A).

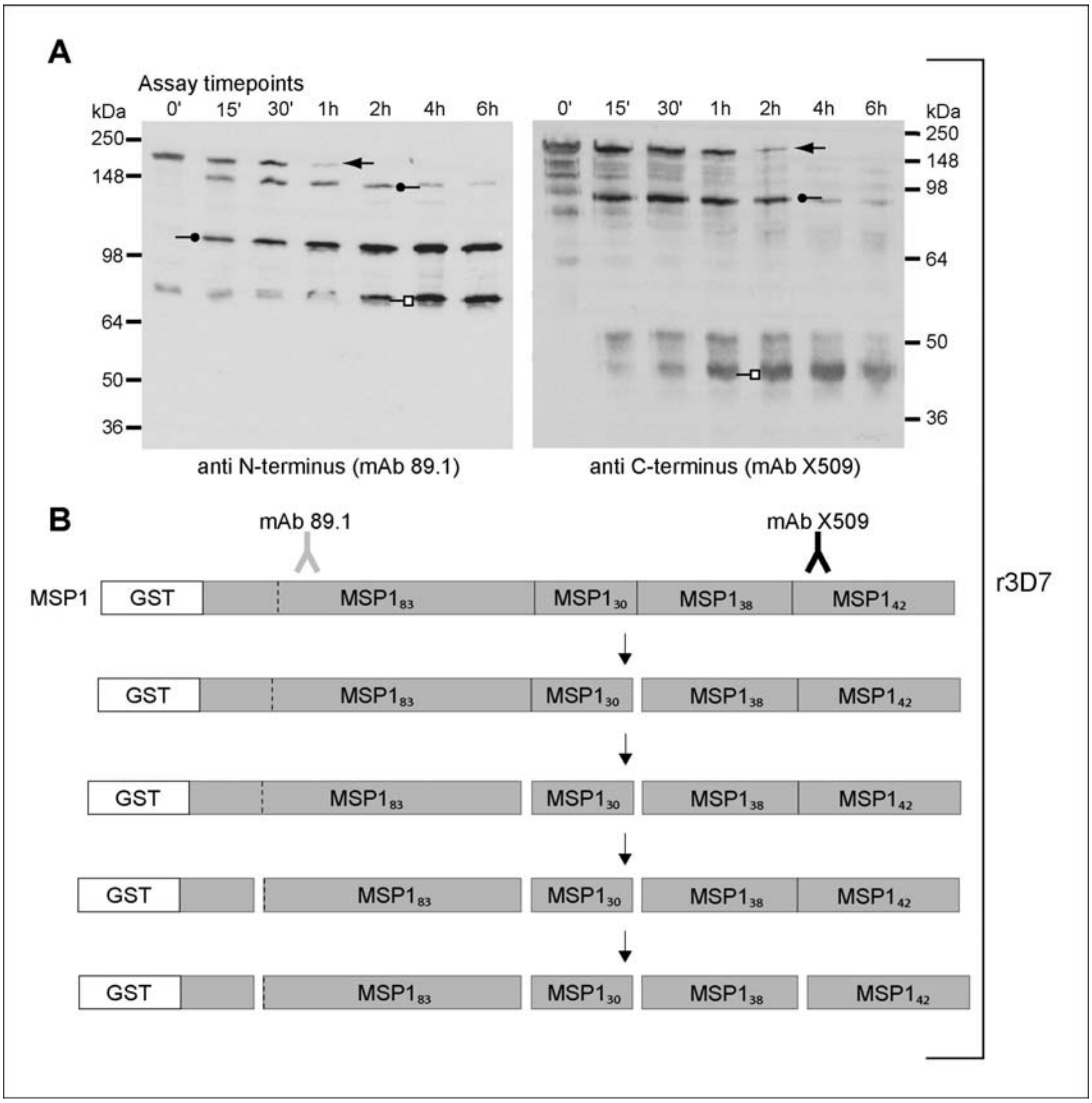

Figure S2. Correct cleavage by rPfSUB1 of a synthetic peptide substrate based on an artificial cleavage site. Analytical RP-HPLC fractionation of mixtures of the predicted artificial PfSUB1 substrate Ac-FISGQSETDH with the 38/42 peptide from either the Wellcome-type (**A**) or the 3D7-type (**B**) MSP1. In each panel, the top chromatogram shows the elution profile of an equimolar mix of the undigested peptides, whilst the lower chromatogram shows the elution profile after partial digestion with rPfSUB1. Identities of major peaks are indicated. The N-terminal product of cleavage of the artificial substrate, Ac-FISGQ (predicted *m/z* 593.29), was identified by electrospray mass spectrometry (measured *m/z* 593.32). The predicted C-terminal product of cleavage (NH_2-SETDH) was not observed in the RP-HPLC elution profile; it was assumed not to bind to the column due to its hydrophilic nature, as previously found for several PfSUB1 peptide substrates (1).

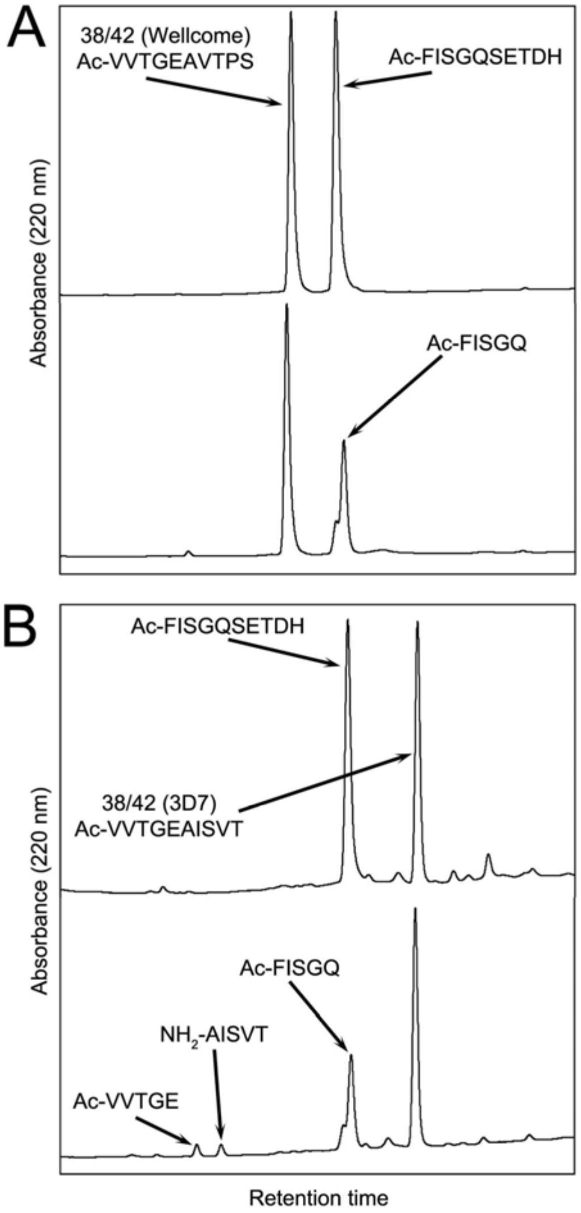

Figure S3. Construction of a chimeric *P. falciparum msp1* gene by homologous recombination. (**A**) The blue highlighted text indicates part of the authentic 3D7 *msp1* sequence used to target constructs pMSP1chimWT and pMSP1chimSUB2to1 for integration. In pMSP1chimWT this was fused to that highlighted in yellow, comprising synthetic, recodonised sequence encoding the C-terminal end of the Wellcome-type MSP1 with its authentic PfSUB2 cleavage site. Integration of pMSP1chimWT was therefore designed to produce a chimeric gene (termed 3D7W), the product of which would be correctly processed by both PfSUB1 and PfSUB2. The synthetic sequence is shown aligned with the corresponding authentic 3D7 *msp1* gene sequence, demonstrating the limited sequence identity at the nucleotide level (asterisks indicate positions of identity), designed to force crossover upstream of sequence encoding the secondary processing site. This is indicated (LQGML↓NISQH), the precise point of cleavage being shown with red arrows. The Wellcome-type specific Gln residue (Q) that lies 14 residues downstream of the cleavage site and is required for recognition by mAb 111.4 is also indicated. (**B**) Integration construct pMSP1chimSUB2to1 differed from pMSP1chimWT only in that the recodonised sequence encoding the PfSUB2 cleavage site was replaced with sequence encoding the PfSUB1-sensitive sequence FISGQSETDH. Shown is an alignment of the chimeric 3D7W sequence predicted to be formed upon integration of pMSP1chimWT, and the chimeric sequence (termed 3D7W-SUB2to1) predicted to be formed upon integration of pMSP1chimSUB2to1. The blue block highlights the secondary processing site encoded by 3D7W-WT, whilst the yellow block highlights the substituted PfSUB1-sensitive sequence encoded by 3D7W-SUB2to1. Predicted positions of cleavage by PfSUB2 and PfSUB1 are indicated.

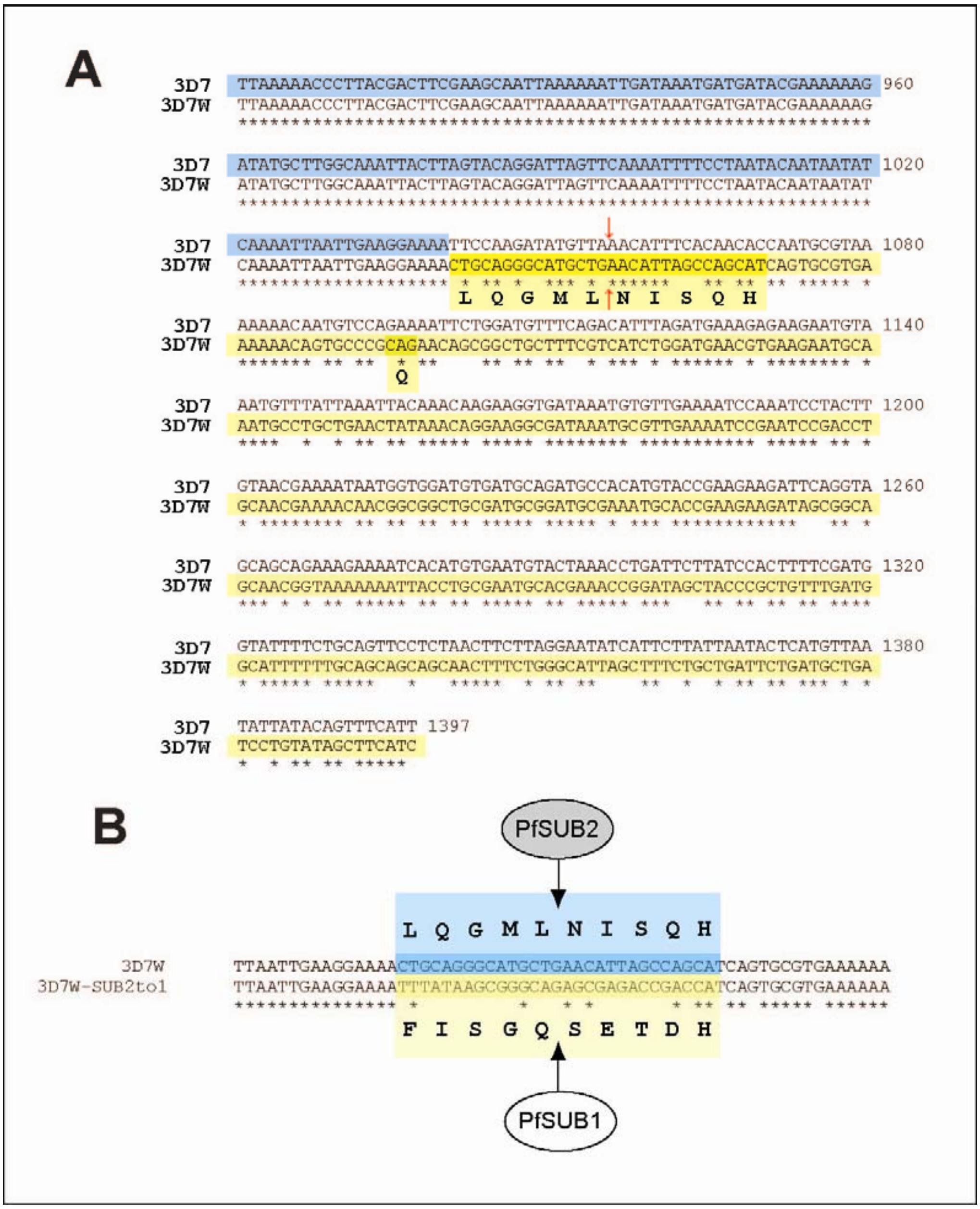

REFERENCES

1. Koussis, K., C. Withers-Martinez, S. Yeoh, M. Child, F. Hackett, E. Knuepfer, L. Juliano, U. Woehlbier, H. Bujard, and M.J. Blackman. 2009. A multifunctional serine protease primes the malaria parasite for red blood cell invasion. *EMBO J* 28:725-735.
